# Serum sclerostin levels in renal cell carcinoma patients with bone metastases

**DOI:** 10.1038/srep33551

**Published:** 2016-09-26

**Authors:** C. Wibmer, K. Amrein, A. Fahrleitner-Pammer, M. M. Gilg, A. Berghold, G. C. Hutterer, W. Maurer-Ertl, A. Gerger, A. Leithner, M. Pichler, J. Szkandera

**Affiliations:** 1 Department of Orthopedic Surgery, Medical University of Graz, Graz, Austria; 2 Department of Medicine, Division of Endocrinology and Diabetology, Medical University of Graz, Graz, Austria; 3 Institute for Medical Informatics, Statistics and Documentation, Medical University of Graz, Graz, Austria; 4 Department of Urology, Medical University of Graz, Graz, Austria; 5 Department of Medicine, Division of Oncology, Medical University of Graz, Graz, Austria; 6 Research Unit Genetic Epidemiology and Pharmacogenetics, Division of Clinical Oncology, Medical University of Graz, Graz, Austria; 7 CB-MED, Medical University of Graz, Graz, Austria; 8 Research Unit for non-coding RNAs and genome editing in cancer, Medical University of Graz, Graz, Austria; 9 Department of Experimental Therapeutics, The University of Texas MD Anderson Cancer Center, Houston, Texas, USA

## Abstract

Sclerostin has been proposed as a potent inhibitor of bone formation. Sclerostin antibodies are under clinical development to treat osteoporosis and metastatic bone disease. Serum sclerostin level is elevated in multiple myeloma, an osteolytic malignancy, where it might serve as predictive marker for the use of sclerostin-directed antibodies. As renal cell carcinoma (RCC) patients often present with osteolytic metastases, we aimed to investigate serum sclerostin levels in RCC patients. Our study included 53 RCC patients (19 with bone metastases, 25 with visceral metastases and 9 with localized disease) and 53 age- and gender-matched non-osteoporotic controls. Frozen serum samples were subjected to sclerostin quantitative sandwich ELISA. The mean serum sclerostin levels of RCC patients and controls were 45.8 pmol/l and 45.1 pmol/l, respectively (p = 0.86). Analysis of variance showed no difference between the subgroups of RCC patients with regard to visceral or bone metastases or localized disease (p = 0.22). There was no significant association between eGFR (estimated glomerular filtration rate) and serum sclerostin levels in RCC patients (r = 0.05; p = 0.74) and controls (r = 0.06; p = 0.68). Our results indicate that serum sclerostin levels appear not to be a valuable biomarker to assess the occurrence of bone metastases in RCC patients.

Sclerostin is a glycoprotein with a C-terminal cysteine-knot-like (CTCK) domain and is encoded by the SOST gene. It has been found to be a potent inhibitor of bone formation^1^,^2^ and is secreted mainly by mature osteocytes^3^. Recently it was also shown to be expressed in chondrocytes in mineralized cartilage^4^. Secretion of sclerostin is downregulated by mechanical loading of bone^5^,^6^,^7^,^8^ and parathyroid hormone (PTH)^9^ and induced by pro-inflammatory cytokines^9^,^10^,^11^,^12^. SOST-knockout mice and patients with homozygous defects for SOST show the same picture of extremely high bone density phenotypes: sclerosteosis and van Buchem disease^1^,^3^. Wijenayaka *et al*.^2^ first described the pathway that underlies the influence of sclerostin on bone formation. They reported that sclerostin inhibits the Wnt/ß-catenin pathway by inhibiting low-density lipoprotein receptor-related protein 5 (Lrp5). This receptor, as a coreceptor of the frizzled family receptors, activates the canonical Wnt/ß-catenin-pathway, thus stimulating the Wnt target genes^2^,^10^. These target genes in turn are osteoblast marker genes that inhibit osteoblast differentiation and proliferation as well as osteoprotegerin mediated effects (OPG). Increased sclerostin signaling is thought to be involved in osteoporosis and a humanized monoclonal sclerostin antibody (AMG785) is currently tested in clinical trials to increase bone formation and decrease bone resorption^13^,^14^,^15^.

Sclerostin antibody has been presented as a possible future therapeutic option for bone metastases^11^,^12^,^16^. In metastatic bone disease (MBD) there is evidence that Wnt-regulating molecules such as sclerostin contribute to the development and progression of osteoblastic and/or osteolytic metastases, depending on the rate of expression^11^,^16^. For multiple myeloma (MM), often presenting with osteolytic lesions, a positive correlation between the severity of bone disease and circulating levels of serum sclerostin has been found, suggesting that MM-cells utilize sclerostin in the formation of osteolytic lesions^17^,^18^.

Renal cell carcinoma (RCC) accounts for approximately 4% of all malignancies in Western Europe and the USA; the age-adjusted incidence rate lies between 6.2-8.4/100,000/year, and the male to female ratio is 3:2^19^,^20^. It occurs less frequently in developing countries^21^. Tobacco smoking and overweight have been identified as major etiological factors^21^. The 5-year survival rate increased significantly to 75% during the last 30 years^19^,^20^. About 30% of the RCC patients develop bone metastases^22^ which are osteolytic and highly vascularized, as well as chemo- and radio-resistant. RCC was the fourth most common cancer presenting with symptomatic bone metastases in our own patient collectives, accounting for seven percent of patients treated for spinal metastases^23^,^24^. Since there are no reports to date on serum sclerostin levels in RCC, we systematically evaluated them in different groups of RCC patients: patients with localized disease, bone or visceral metastases and compared them to serum sclerostin levels in non-osteoporotic controls.

## Methods

This case-control study included 53 patients with histologically confirmed RCC treated at the Division of Oncology, Medical University of Graz between 1986 and 2012. All included RCC patients with bone or visceral metastases had a minimum follow-up of 12 months if alive with disease. For the patients with localized disease a minimum follow-up of 4 years was required for inclusion in this study. Clinico-pathological data were retrieved from medical records at the Division of Oncology. Bone metastases were confirmed either by histology, or characteristic magnetic resonance imaging (MRI), computed tomography (CT), or bone scan with multiple lesions; visceral metastases were also confirmed by imaging techniques (MRI or CT). All patients were routinely followed at the Division of Oncology, Medical University of Graz, with examinations at regular intervals (three-month intervals for the first two years after diagnosis, then six-month intervals for years two to five, 12-month intervals for years 6–10). The serum samples were taken within 90 days after diagnosis of primary disease or bone or visceral metastasis, respectively.

The controls were matched for age and gender as recent studies have demonstrated that sclerostin levels are age dependent^25^,^26^. Controls were either blood donors (age under 65 years) or non-osteoporotic patients with osteoarthritis (OA) (age over 65 years), as no difference in sclerostin levels has been reported between patients with OA and healthy controls^27^. The controls with OA were treated at the Department of Orthopedics and Orthopedic Surgery, Medical University of Graz and the clinical and demographic data were obtained from their medical records.

Exclusion criteria for the RCC patients were primary diagnosis of RCC before 1986, further malignant disease other than RCC and osteoporosis. Exclusion criteria for the controls were osteoporosis, malignant disease and chronic kidney disease. Furthermore, all patients and controls with concomitant use of drugs potentially interfering with serum sclerostin levels, such as glucocorticoids, bisphosphonates and denosumab^28^,^29^,^30^,^31^,^32^, were excluded from our study. Renal function was estimated using serum creatinine and the CKD-EPI creatinine equation^33^. As reduced renal function is common in RCC patients, usually following tumor nephrectomy, we analyzed the correlation between eGFR (estimated glomerular filtration rate) and serum sclerostin levels. The participants had no dietary restrictions. The samples used for this research project were kindly provided by Biobank Graz (RCC patients and patients with OA) and the Division of Endocrinology and Diabetology, Department of Internal Medicine, Medical University of Graz (blood donors).

Sclerostin levels in blood serum samples (stored at −70 °C) were determined with a quantitative sandwich ELISA (Sclerostin ELISA, Biomedica, Vienna, Austria) according to the standard protocol provided by the manufacturer. Intra- and interassay coefficients of variation were <7% and <10%, respectively. All samples were assayed in duplicate.

This study and its experimental protocol were approved by the ethics committee of the Medical University of Graz (EK-Nr: 18-271 ex 06/07, amendment 2, of 22 July, 2013) IRB00002556 (Institutional Review Board Registry) and the methods and all experiments were performed in accordance with relevant guidelines and regulations. Informed consent was obtained from all subjects before sample-taking.

### Statistical analysis

The data were first analyzed with descriptive statistics, using mean and standard deviation for continuous data like age and laboratory parameters. Categorical data are described by absolute frequencies. The differences in serum sclerostin levels between RCC patients and controls were analyzed with the t-test and ANOVA. Pearson’s correlation coefficient was calculated for eGFR and serum sclerostin values. All analyses were performed with the statistical software package IBM SPSS Statistics version 23. A two-sided p-value less than 5% was considered significant.

## Results

Our study included 9 patients with localized disease, 19 patients with bone metastases and 25 patients with visceral metastases only (no bone metastases). The male to female ratio was approximately 2:1, the mean age 62 years (range 31 to 89). At last follow-up (July 11, 2016) 70% of all included RCC patients had died, in detail: 22% of the RCC patients with localized disease and 84% of the RCC patients with metastases (visceral or bone metastases). Table 1 summarizes the clinical characteristics and laboratory data of the RCC patients and controls.

**Figure 1:**
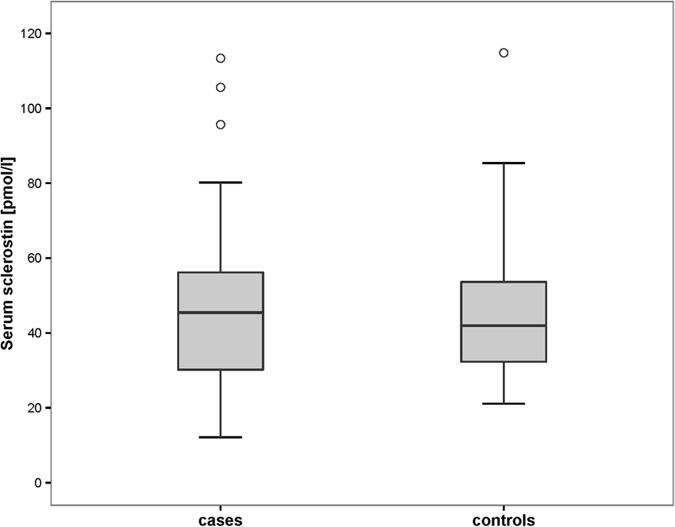
Serum sclerostin levels in RCC patients (cases) and controls (controls) (p = 0.86).

**Figure 2:**
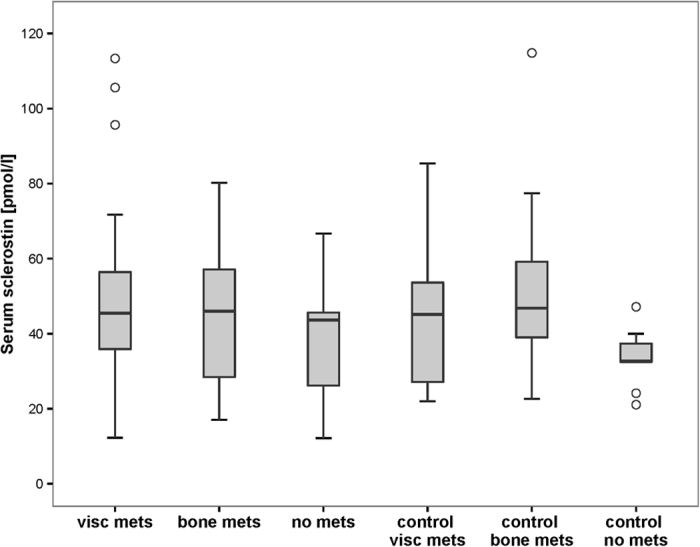
Serum sclerostin levels in the subgroups of RCC patients and corresponding controls (p = 0.22).

The mean serum sclerostin levels of RCC patients and controls were 45.8 pmol/l (SD 22.2) and 45.1 pmol/l (SD 18.9) respectively (Table 2). There was no difference between the serum sclerostin levels of RCC patients and controls (p = 0.86) (Fig. 1). The mean serum sclerostin level was 37.2 pmol/l (SD 17.8) in RCC patients with localized disease, 44.8 pmol/l (SD 18.5) in RCC patients with bone metastases and 49.7 pmol/l (SD 25.7) in RCC patients with visceral metastases. The corresponding control groups showed serum sclerostin levels of 33.7 pmol/l (SD 7.9), 51.3 pmol/l (SD 21.3) and 44.6 pmol/l (SD 18.3) respectively. The analysis of variance showed no difference between the subgroups of RCC patients with visceral or bone metastases or localized disease and their corresponding controls (p = 0.22) (Table 2, Fig. 2).

**Figure 3:**
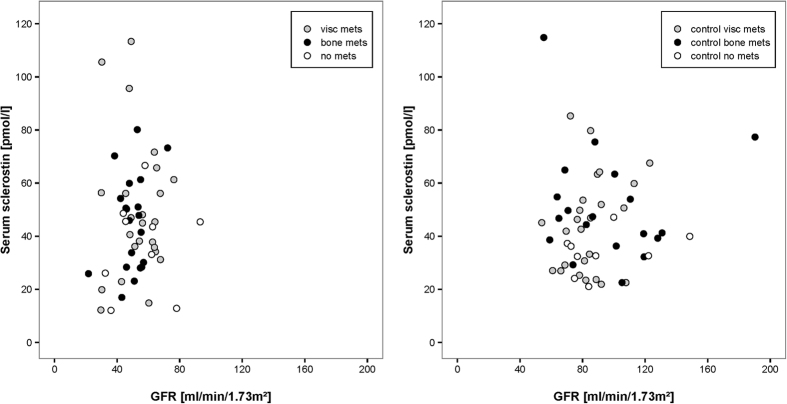
Correlation between serum sclerostin and GFR in RCC patients (left; r = 0.05, p = 0.74) and controls (right; r = 0.06, p = 0.68). Visc mets…RCC patients with visceral metastases, bone mets…RCC patients with bone metastases, no mets…RCC patients with localized disease, contr visc mets…healthy controls for RCC patients with visceral metastases, contr bone mets…healthy controls for RCC patients with bone metastases, contr no mets…healthy controls for RCC patients with localized disease.

With a mean eGFR of 52.4 ml/min/1.73 m^2^ (SD 13.5) the RCC patients had distinctly lower values than the controls (89.8 ml/min/1.73 m^2^) (SD 25.2) (Table 1). Pearson´s correlation analysis showed no significant association between eGFR and serum sclerostin levels in RCC patients (r = 0.05; p = 0.74) and controls (r = 0.06; p = 0.68) (Fig. 3).

## Discussion

In recent reports, it has been hypothesized that sclerostin might be a possible target for therapeutic agents in prostate and breast cancer patients with bone metastases^11^,^12^,^16^. Bone metastases can be osteoblastic or osteolytic^16^; both may lead to acute and chronic pain, but with osteolytic metastases there is also a higher risk of pathologic fracture^11^. Besides the common anti-resorptive agents such as bisphosphonates and the RANKL-inhibitor Denosumab, new treatment options with stimulators of bone formation are under intensive investigation^11^, in the hope of achieving a better bone metabolism balance. A sclerostin-directed antibody as a treatment option for bone metastases would address such an osteoanabolic approach. Since RCC patients frequently develop osteolytic bone metastases^22^ and many of them require orthopedic-oncological surgery for pathological or impending fractures, we examined whether serum sclerostin levels in RCC patients are different from healthy controls and whether osteolytic bone metastases are associated with higher sclerostin serum levels. In this study, we found neither significant differences between serum sclerostin levels in RCC patients and controls, nor different sclerostin levels in RCC patients with bone versus non-bone metastases. Interestingly, the opposite has been reported for MM, which also presents with osteolytic lesions. In this entity elevated serum sclerostin levels and even a correlation between sclerostin expression in MM-cells and severity of disease, have been found^17^. Elevated serum sclerostin levels were also reported in prostate cancer, but without an analysis of bone metastases^34^. The role of sclerostin in cancer and SOSTDC1 (sclerostin domain containing 1)-expression in cancerous tissue was addressed in several studies. A downregulation of SOSTDC1 was found in breast cancer and RCC tissues^35^,^36^. Additionally, immunochemistry showed lower sclerostin expression in prostate cancer than in normal prostate tissue^37^. It is likewise reported that prostate and breast cancer cells produce DKK-1^38^,^39^ which, like sclerostin, inhibits Lrp5 (activator of the Wnt/ß-catenin pathway). An *in vitro* three dimensional model of metastastic bone formation has been developed to study microenvironmental interactions with breast cancer cells^40^. This model showed a reduction in osteoblastic tissue thickness and an increase in osteoclastogenesis in the presence of breast cancer cells^41^. Even though these models do not yet provide detailed information on sclerostin, they seem to show promise for clarifying the underlying pathomechanism between sclerostin and metastatic bone disease.

If and how sclerostin antibody will represent a therapy option in bone metastases in RCC patients is still unresolved. Our results imply that serum sclerostin levels are not suitable to detect bone metastases in RCC patients and that increased levels of sclerostin cannot be used as a potentially predictive marker for sclerostin-directed treatment strategies.

Our study population of RCC patients had lower eGFR-levels than the controls, mainly due to previous nephrectomy. Data on the correlation of serum sclerostin and chronic kidney disease (CDK) is scarce and controversially reported. Most of the published data report elevated serum sclerostin levels with declining kidney function^42^,^43^,^44^, but other studies found elevated renal elimination of sclerostin in CKD^45^ and reduced serum sclerostin in children with CKD^46^. In our study, we found no significant association between eGFR and serum sclerostin levels in RCC patients and controls.

Recent reports demonstrated that elevated serum sclerostin levels correlate with high bone turnover^42^, increasing age^25^,^47^,^48^ and high ratio of fat mass as well as bone mass^49^,^50^. On the other hand, serum sclerostin levels decrease with greater physical activity^51^. Results differ on changes in serum sclerostin levels in relation to osteoporosis. Ardawi *et al*. suggest that serum sclerostin levels predict the risk of osteoporosis related fractures (ORF), reporting a sevenfold higher risk for ORF for each standard deviation of the sclerostin level above the normal range^26^. This is in contrast to a study that compared serum sclerostin levels in healthy controls and/or patients with osteoporosis to patients with OA^27^ where no significant difference was found (0.78 vs 0.71 vs 0.80 ng/ml) between these groups, as well as to the findings of Amrein *et al*. that serum sclerostin levels do not linearly correlate with fracture risk^52^. As the correlation of serum sclerostin levels to sclerostin expression in bone and cancer tissue, and the role of sclerostin in different context are not thoroughly clear and, similarly, the effects of CKD on serum sclerostin levels are uncertain, we conclude that serum sclerostin levels have to be interpreted with caution and are currently not a useful biomarker in this setting.

We acknowledge the limitations of this study, mostly arising from the retrospective design and the small number of cases, as well as the absence of data on physical activity, which might bias the results.

## Conclusions

As we found no significant differences between the serum sclerostin levels in patients with RCC and controls, sclerostin serum level appears not to be a useful biomarker for assessing bone metastases in RCC patients. Furthermore, other approaches have to be explored to select RCC-patients, who might benefit from sclerostin antibodies as a further therapy option for bone metastases.

## Additional Information

**How to cite this article**: Wibmer, C. *et al*. Serum sclerostin levels in renal cell carcinoma patients with bone metastases. *Sci. Rep*. **6**, 33551; doi: 10.1038/srep33551 (2016).

## Figures and Tables

**Table 1:** Patient characteristics and laboratory results.

		Gender m:f	Age [years] (min/max)	eGFR [ml/min/1.73 m^2^] (SD)
total	RCC patients	36:17	63 (31/89)	52.8 (13.2)
	Controls	36:17	64 (33/89)	89.8 (25.2)
subgroups	Visc mets	16:9	66 (41/83)	53.9 (13.2)
	Bone mets	15:4	62 (31/89)	49.6 (9.9)
	No mets	5:4	58 (42/77)	56.8 (19.8)
	Contr visc mets	16:9	66 (41/83)	84.1 (16.0)
	Contr bone mets	15:4	62 (33/89)	95.7 (33.2)
	Contr no mets	5:4	58 (42/77)	93.0 (26.5)

Data are presented as mean, SD…standard deviation, eGFR…estimated GFR (CKD-EPI), visc mets…RCC patients with visceral metastases, bone mets…RCC patients with bone metastases, no mets…RCC patients with localized disease, contr visc mets…healthy controls for RCC patients with visceral metastases, contr bone mets…healthy controls for RCC patients with bone metastases, contr no mets…healthy controls for RCC patients with localized disease.

**Table 2:** Serum sclerostin levels in RCC patients with visceral metastases, bone metastases and localized disease and age and gender-adjusted controls.

		Serum sclerostin [pmol/l] (SD)	p-value
total	RCC patients	45.8 (22.2)	0.86#
	Controls	45.1 (18.9)	
subgroups	Visc mets	49.7 (25.7)	0.22§
	Bone mets	44.8 (18.5)	
	No mets	37.2 (17.8)	
	Contr visc mets	44.6 (18.3)	
	Contr bone mets	51.3 (21.3)	
	Contr no mets	33.7 (7.9)	

^#^Comparison of RCC patients and controls using t-Test,

^§^using ANOVA.
